# Niche Segregation between Wild and Domestic Herbivores in Chilean Patagonia

**DOI:** 10.1371/journal.pone.0059326

**Published:** 2013-03-21

**Authors:** Esperanza C. Iranzo, Juan Traba, Pablo Acebes, Benito A. González, Cristina Mata, Cristián F. Estades, Juan E. Malo

**Affiliations:** 1 Terrestrial Ecology Group-TEG, Departamento de Ecología, Facultad de Ciencias, Universidad Autónoma de Madrid, Madrid, Spain; 2 Departamento de Manejo de Recursos Forestales, Facultad de Ciencias Forestales y de la Conservación de la Naturaleza, Universidad de Chile, Santiago, Chile; University of Western Ontario, Canada

## Abstract

Competition arises when two co-occuring species share a limiting resource. Potential for competition is higher when species have coexisted for a short time, as it is the case for herbivores and livestock introduced in natural systems. Sheep, introduced in the late 19^th^ century in Patagonia, bear a great resemblance in size and diet to the guanaco, the main native herbivore in Patagonia. In such circumstances, it could be expected that the two species compete and one of them could be displaced. We investigated spatial overlap and habitat selection by coexisting sheep and guanaco in winter and in summer. Additionally, we studied habitat selection of the guanaco in a control situation free from sheep, both in summer and winter. We also determined overlap between species in areas with different intensity of use (named preferred and marginal areas) in order to further detect the potential level of competition in the case of overlapping. Guanaco and sheep showed significantly different habitat preferences through all seasons, in spite of their spatial overlap at landscape scale. Additionally, the habitat used by guanaco was similar regardless of the presence or absence of livestock, which further indicates that sheep is not displacing guanaco where they coexist. These results suggest that habitat segregation between guanaco and sheep is due to a differential habitat selection and not to a competitive displacement process. Therefore, the potential for competition is considered low, contrary to what has been previously observed, although this could be a density-dependent result.

## Introduction

Animal community structure results from multiple interactions among biotic and abiotic factors that determine different species habitat selection [1], [2], [3]. Resource availability, distribution and quality are essential factors to explain spatial distribution of species [3], [4]. In addition, for large herbivores, key habitat-selection factors include interspecific competition [5], [6] and predation risk [7].

Interactions between large herbivores are not easy to detect, measure and interpret [1], [4], [8]. Interspecific competition can occur by interference (direct competition) or by exploitation of the same resources (indirect competition [1], [4]). Furthermore, spatial and temporal scales in which such interactions take place usually difficult their study, especially in the absence of control situations and/or when variable species densities are involved [4], [9], [10].

According to ecological theory, two species compete when they overlap in their use of limiting spatial and trophic resources [1], [11], [12]. When species coexist through evolutionary time, resource partitioning mechanisms can evolve to minimize competition and, thus, to enable coexistence [5], [11], [12]. However, the recent introduction of domestic species into an original assemblage may trigger processes of interspecific competition with native ones, although the intensity of these processes depends on the level of overlap between species and the generalist or specialist character of them [4], [13], [14]. When competition occurs, it is expected to be more severe between species with similar foraging strategy and size [4], and it is expected to trigger changes in the patterns of resource use of one or all of the species involved at the cost of a partial displacement away from their optimum [1], [6], [11]. In these cases, animals may be lead to occupy habitats different to the preferred ones (sub-optimal habitats). In addition, competing species may show an apparent lack of competition due to competitive exclusion, often resulting in ambiguous spatial patterns [15]. Conflict between sympatric species can increase when man favours one of them, which is the case where wild species coexist with domestic livestock [8], [13], [16]. In such cases, populations of native species can suffer negative impacts ranging from competitive displacement to poorer areas, demographic effects, or even local extinction [8], [16], [17], [18]. Besides, in these circumstances it is difficult to tease apart the direct effect of livestock on the native herbivore from that of human management of the grazing area.

The guanaco (*Lama guanicoe*) is the only large native herbivore widely distributed throughout Patagonia. Since the introduction of sheep in the late 19^th^ century, this species has suffered a dramatic population decline attributed to competition with livestock, poaching and habitat degradation and fragmentation [19], [20], [21]. Currently, the IUCN estimates a total guanaco population of about 600000 individuals, of which 9% are in Chile [21]. Meanwhile, the number of sheep in Patagonia increased rapidly, reaching 22 million heads in 1950 [22]. Today there are yet about 4 million sheep under an extensive free grazing system, being a study case for potential competition with guanaco.

Guanaco has been described as a generalist herbivore that shows preference for grasslands and open ranges with short vegetation. Its diet varies along its geographic distribution and it has been characterized as a mixed feeder [19], [20], [23], [24]. Sheep is also characterized as a generalist herbivore which shows some preference for grasses [25]. Previous studies in Patagonia have shown that both species overlap in their niches [14], [19] and exhibit high similarity in the composition of their diets (up to 80%), in fact, two grass species constitute the 40% of both guanaco and sheep diets [14], [23], [25], [26]. These facts point to a high potential for competition in places where both species coexist [19], [25]. To unravel this question it could be useful to investigate how habitat selection of native species varies in the presence and absence of the introduced one. For this objective, the presence of protected areas without livestock within a matrix of ranching areas in which both species coexist arises as an ideal natural field experiment.

The aim of this paper is to evaluate habitat selection and the degree of overlap between two recently sympatric species: guanaco and sheep. The study area allowed to analyse species coexistence in an environment inhabited by both species (hereafter non-protected area) and to compare these results with an adjacent control area where only the guanaco is present (Torres del Paine National Park, hereafter TPNP). This, therefore, will allow to accurately assess the habitat preferences of the native herbivore in the absence of the introduced one. On the framework of competition and coexistence developed above, we discuss the recent entry of a domestic herbivore in a native assemblage, given high similarity in size, diet and requirements to the native species (guanaco) following next premises: 1) in the absence of sheep, inside the TPNP, the guanaco will select open areas with low-size vegetation; 2) where both species coexist, in the non-protected area, the guanaco will modify its habitat selection towards less preferred areas with regard to those in TPNP. In this case, it is expected to find segregation between preferred areas for both species, but some overlap between marginal and preferred areas may be detected.

## Materials and Methods

### Ethics Statement

The present study did not need the capture or handling of protected or endangered animals. All data about species’ locations were collected by observation at distance using binoculars. The described field studies were carried out on a protected area and on privately-owned farms with the permission of both, CONAF (Corporación Nacional Forestal de Chile) and farmers.

### Study Area

**Figure 1:**
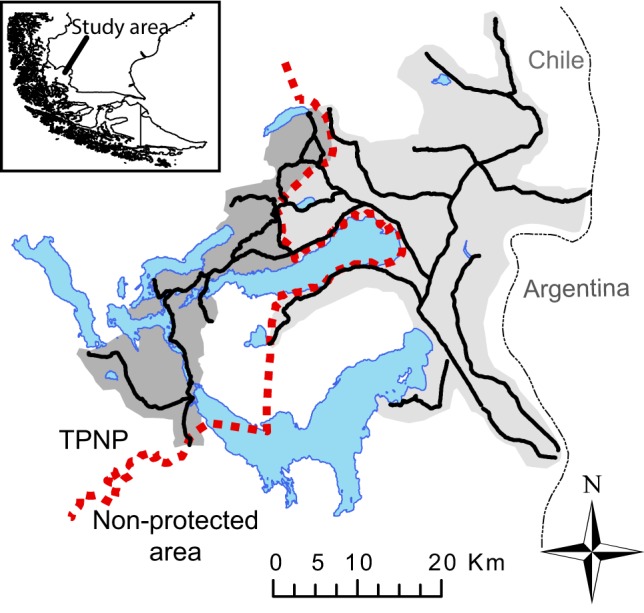
Location of study area (shaded area) in the Comuna Torres del Paine (Region of Magallanes, Chile). Red dashed line indicates Torres del Paine National Park (TPNP) boundary. Black solid lines represent the surveyed road network.

The study was carried out inside and around the Torres del Paine National Park (51°3’S 72°55’W, Última Esperanza province, Region of Magallanes, Chile; Fig. 1), particularly in an area of 1090 km^2^ (284 km^2^ inside the TPNP and 806 km^2^ of the neighbouring farms). Study area belongs to the transition zone forest-steppe. According to the Köppen climate classification system, climate is temperate-cool without dry season. Annual rainfall varies between 300 and 1000 mm, mean temperature ranging from 2°C in winter to 10.8°C in summer. This study differentiated 10 habitats types (Table 1), most of them defined by plant communities [27], [28].

**Table 1:** Variables recorded for each guanaco (*Lama guanicoe*) and sheep (*Ovis aries*) sighting, and to estimate habitat availability at each control point.

	Variable	Description
Topographic	Slope	Three levels: low (<5%), medium (5–45%), high (>45%)
	Topographic position	Four levels: valley bottom, lower half of hillside, upper half of hillside, peak
	Roughness	Three levels: low (good visibility), medium (reduced visibility in some direction), high (low visibility)
Physiognomy	Mean vegetation height	Medium vegetation height in cm
	Maximum vegetation height	Maximum vegetation height in cm
Habitat	Water	Bodies of running water (rivers or streams) or stagnant water (lakes and ponds)
	Bare soil	Without vegetation ground cover or rocks
	Natural grassland	Unmanaged grasslands dominated by native herbaceous species
	Managed grassland	Grasslands sown with fodder species
	Coironal	Steppe-like grasslands dominated by tufted grasses from genera Festuca and Stipa
	Wetland	Vegetation of shallow wetlands or flooded depressions with predominance of genera Carex and Juncus
	Xerophytic scrub	Small-sized scrubs, forming cushions and adapted to water deficit and strong winds. The most frequent species are *Mulinum spinosum* and *Senecio patagonicus*
	Mata Negra	Midheight woody shrub. This species (*Junellia tridens*) forms dense communities
	Mesophytic scrub	Robust scrubs reaching 4 m in height. Characterized by *Berberis microphylla* that require higher precipitation and wind protection
	Woods	Lenga *(Nothofagus pumilio)* and/or Ñirre (*Nothofagus Antarctica)* forest patches

Each variable was measured for a 50 m radius from the animal or the centroid of the group.

TPNP is only inhabited by wild animal populations, guanaco is the most abundant herbivore and hare (*Lepus europaeus*) and upland goose (*Chloephaga picta*) are present with lower densities. Rhea (*Pterocnemia pennata*) and huemul (*Hippocamelus bisulcus*) are also present but rare. The TPNP surroundings are private owned lands dedicated to extensive livestock farming, mainly sheep but also cattle and horses. Sheep graze freely in large pasture lots of several square kilometers without the continuous presence of shepherds. They are moved between pasture lots twice a year and shepherds occasionally visit flocks to verify that the animals are in good condition. In this area, livestock coexists with wild guanaco populations and other less abundant herbivore species such as rhea, upland goose or hare.

### Data Collection

Sampling of herbivores was conducted during the winters and summers of 2009 and 2010, corresponding with times of minimum and maximum abundance of trophic resources, respectively. In each of the four sampling seasons, all existing roads and paths in the TPNP were travelled by vehicle or on foot (N = 12; 76.6 km), as well as those in the non-protected area (N = 17; 221.8 km). Both methods are considered comparable since they do not disturb animal behaviour or habitat selection in the study area, where animals show short flight distances [29]. Sampling was carried out during daylight hours, avoiding sunrise and sunset. For each animal or group of animals encountered the centroid of the group was located and the point was recorded with a GPS. Considering a 50 m radius around the centroid, the following descriptors were recorded: habitat composition (coverage (%) of different vegetation types), topography and physiognomy of the location of the sighting (Table 1).

To determine habitat availability, the same roads and paths were travelled in winter and summer, and the environmental variables were measured independently of the presence of animals. Two control sampling points (pseudo-absences) were located every 1000 m, at 100 m and 250 m on each side of the road alternately (N = 194 sampling points in TPNP, N = 394 sampling points in non-protected area in winter; N = 222 sampling points in TPNP and N = 366 points in non-protected area in summer). At each sampling point a plot of 50 m radius was established and the same variables described for herbivore sightings were measured (Table 1). Control plots have been classified as either inside or outside TPNP to take account in the analyses for differences in habitat availability between areas.

### Data Analysis

Firstly, we performed a point pattern analysis for each season and year in order to analyze the spatial pattern of landscape use by both species and detect their spatial overlap at intermediate (landscape) scale. We used a mathematic transformation of K-Ripley analysis, the bivariate function L(r), in order to test the spatial aggregation between guanaco and sheep locations at scales from 0 to 2000 m in the non-protected area. If there is spatial overlap at that scale, there is some potential for competition. These analyses were performed with Passage software version 2 [30].

Habitat selection analysis at a fine scale was performed in several sequential steps on habitat variables measured at a 50 m radius scale for each observation. Firstly, general discriminant analyses (GDA) were used to determine whether the habitats used by both species were different from each another and from the availability, or whether guanaco and sheep showed overlap in resource use by selecting similar habitats. GDA combines predictor variables on a reduced number of axes, orthogonal to each other, and allows the detection of differences between a priori defined groups (see below). These axes can be interpreted as niche dimensions, as they include information of original variables related not only to habitat used by animals but also to habitat availability. To test the relative weight of each axis to the overall discrimination power of the model, 1-Wilks’ λ statistic was used. Values of 1-Wilks’ λ indicate the discriminatory power of models in the range of 1 (perfect discrimination) to 0 (no discrimination) for the whole model as well as for the sub-models obtained after removing the respective axis. Analyses were performed separately for each season in order to detect temporal overlap in habitats used. To assess differences in habitat selection of guanaco attributable to the presence of sheep in the non-protected area, guanaco observations inside and outside TPNP were treated independently, thus considering 5 different groups for analyses: Habitat availability (controls in TPNP/controls in non-protected area), guanaco (TPNP/non-protected area) and sheep (just in non-protected area).

Complementarily, to test which groups differed from others in their position on the discriminant axes, a MANOVA test was performed for each season using the coordinates on the canonical axes to define the multivariate space of the test and the group as a factor. Differences between groups were determined by post-hoc Unequal-N HSD tests. These analyses were performed with STATISTICA 8 [31].

In order to detect differences in the overlap between guanaco and sheep related to preferred areas (defined as habitats of maximum utilization) and marginal areas (defined as habitats of marginal use), an analysis based on kernel functions was conducted on the points (observations) within the two-dimensional space defined by GDA axes. This analysis follows the assumption that animals can occupy sub-optimal or less preferred areas where they are more liable to overlap with the other species. Preferred and marginal areas were determined for guanacos and sheep in the non-protected area using an adaptive kernel [32] from the coordinates for each species in discriminant axes. The kernel density estimator is used to describe the intensity of use on a two-dimensional representation of the relative frequency distribution of animals’ locations over a specified period of time [33]. Therefore, it is a good estimator of preferred areas, since it minimizes the influence of isolated points [34]. Kernel estimates can be visualized as a sum of ‘bumps’ placed over the individual locations, so that the density estimate will show large bumps in areas of the GDA space where concentrations of points occur [32]. Preferred areas were calculated by the commonly used kernel 50% isopleth [32], [35] and sightings within it defined as of preferred area. The remaining sightings of each species were classified as occupying marginal areas. ArcView 3.3 [36] was used to define preferred and marginal areas of each species. To determine whether preferred areas of both species overlapped, new MANOVA and post-hoc Unequal-N HSD tests on the coordinates of the canonical axis were performed for each season and year using species as factor. Similar tests were performed to detect overlap between marginal areas and between preferred and marginal ones. These analyses were performed with STATISTICA 8 [31].

## Results

Throughout the four seasons 1446 groups of guanacos were sighted (N_TPNP_ = 550, N_non-protected area_ = 896) totalling 20958 individuals (N_TPNP_ = 7938, N_non-protected area_ = 13020), and 561 groups of sheep (82339 animals).

**Figure 2:**
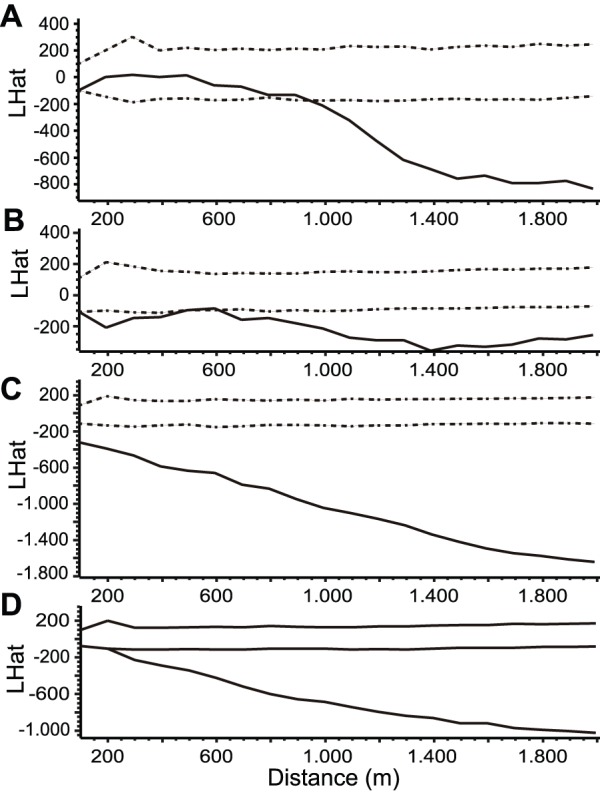
Spatial pattern of aggregation between guanaco and sheep in non-protected areas up to 2000 m. Bivariate function L(r) calculated for (A) winter 2009; (B) summer 2009; (C) winter 2010; (D) summer 2010. The dotted lines represent the 95% confidence interval of a standard complete spatial randomness of the same intensity. For this representation, values below the confidence interval indicate aggregation between guanaco and sheep, while higher values indicate segregation.

Point pattern analyses showed an aggregated pattern between guanaco and sheep for three of the four seasons (summer 2009, winter 2010 and summer 2010) at spatial scales lesser than 500 m. During winter 2009, both species showed a random pattern at these scales (Fig. 2).

In relation with the GDA analyses, the whole models discriminated clearly between groups, both in winter and in summer. According to Wilks’ λ, in both seasons the discriminatory power of the models (defined as 1-Wilks’ λ) was largely determined by the first two axes (Table 2), while the remaining axes achieved an average of 9–12% of discriminatory information present in habitat variables.

**Table 2:** Main results of GDA per season to test the presence of differences in habitat variables among groups.

Season	Model	Discriminatorypower(1- Wilks’ λ)	P-level
Winter	whole model	0.393	<0.001
	after removal of axis 1	0.206	<0.001
	after removal of axes 1 and 2	0.130	<0.001
Summer	whole model	0.463	<0.001
	after removal of axis 1	0.264	<0.001
	after removal of axes 1 and 2	0.191	<0.001

Results of general discriminant analysis (GDA) per season to test whether groups of observations (i.e. availability in Torres del Paine National Park and in non-protected area, and sites selected by guanaco and sheep) could be distinguished on the basis of habitat variables. Values of 1-Wilks’ λ indicate the discriminatory power of models in the range of 1 (perfect discrimination) to 0 (no discrimination) for the whole model as well as for the sub-models obtained by removing the respective axis.

### Winter

In winter, habitats used by both species and habitat availability were differentiated by the full set of discriminant axes (canonical r = 0.486; p<0.001). Of the five significant axes, the first two included 67% of the information contained in habitat variables (Table 2), so that subsequent analyses were based on them. The most important variables to discriminate between groups were topographic position (valley bottom), roughness (low), xerophytic scrub cover, mata negra and bare soil for the first axis; and slope (flat), topographic position (valley bottom) and cover of water, xerophytic scrub and woods for the second (Table 3). The first axis discriminated between sites used by guanaco and habitat availability inside the TPNP, and the sites used by sheep and habitat availability in the non-protected area (F_MANOVA_ = 55.38; p<0.001). The second axis separated habitat availability within the TPNP from everything else (F_MANOVA_ = 17.11; p<0.001).

**Table 3:** Matrix of structure coefficients for discriminant axes in each season.

	Winter		Summer		
	Axis 1	Axis 2	Axis 1		Axis 2
Slope (low)	0.376	−0.375	−0.510	−0.185	
Slope (medium)	−0.116	0.191	0.267	0.238	
Topographic Position (valley bottom)	0.438	−0.311	−0.359	−0.222	
Topographic Position (lower hillside)	0.062	0.224	−0.114	−0.221	
Topographic Position (upper hillside)	−0.143	0.215	0.194	0.106	
Roughness (low)	0.577	0.112	−0.463	−0.237	
Roughness (medium)	−0.266	0.230	0.198	0.019	
Mean vegetation height	−0.029	−0.153	−0.083	0.487	
Maximum vegetation height	0.008	−0.236	−0.051	0.141	
Water	−0.071	−0.496	−0.040	0.256	
Ground cover	−0.513	−0.056	0.393	0.072	
Natural grassland	0.314	0.117	−0.326	−0.083	
Managed grassland	0.119	−0.029	−0.169	0.190	
Coironal	0.192	−0.109	−0.290	−0.167	
Wetland	−0.012	−0.274	0.010	−0.459	
Xerophytic scrub	−0.568	0.493	0.696	−0.017	
Mata Negra	0.424	0.063	−0.381	0.353	
Mesophytic scrub	−0.056	−0.171	−0.024	0.085	
Woods	−0.115	−0.441	−0.036	0.383	

Absolute values indicate correlation of predictor variables with the respective discriminant axes.

**Figure 3:**
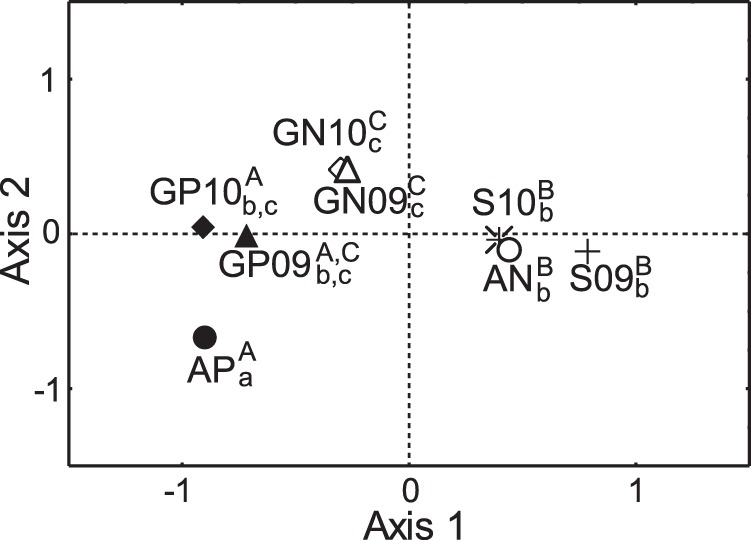
Results of the General Discriminant Analysis (GDA) in winter. Centroids of the observation groups are shown in the space defined by the first two discriminant axes of GDA. AP: Habitat availability in Torres del Paine National Park (TPNP); AN: Habitat availability in non-protected area; GP09: guanaco in TPNP in 2009; GP10: guanaco in TPNP in 2010; GN09: guanaco in non-protected area in 2009; GN10: guanaco in non-protected area in 2010; S09: sheep in non-protected area in 2009; S10: sheep in non-protected area in 2010. Different letters indicate significant differences between groups in first (superscript) and second axis (subscript) in MANOVA analysis according to the Unequal-N HSD *post-hoc* test (P<0.05).

Habitat availability inside TPNP and in the non-protected area (expressed in terms of control sampling points in each zone; Table 4) differed significantly on both axes (Fig. 3). Sites used by sheep could not be discriminated from habitat availability in non-protected area on any of the axes or years (Fig. 3). However, they were segregated from the sites selected by guanaco in the non-protected area in both axes and years, and from those used by guanaco inside the TPNP on axis 1 (Fig. 3). Guanacos in TPNP and in non-protected area did not show significant differences on any axis in 2009 or on the first axis in 2010 (Fig. 3). Lastly, inside the TPNP, sites selected by guanacos were different from availability only on the second axis (Fig. 3), while guanacos in the non-protected area showed significant differences with habitat availability in that area on both axes (Fig. 3).

**Table 4:** Seasonal habitat availability in Torres del Paine National Park and in the non-protected area.

	Winter		Summer	
	TPNP	Non-protected area	TPNP	Non-protected area
Slope (classes 1–3)	1.68	1.45	1.73	1.42
Topographic position (classes 1–4)	2.27	1.91	2.14	1.85
Roughness (classes 1–3)	1.66	1.20	1.64	1.22
Mean vegetation height (cm)	33.4	29.7	37.6	39.4
Maximum vegetation height (cm)	93.0	80.9	107.5	101.1
Water (%)	6.83	2.70	2.64	1.23
Ground cover (%)	15.83	6.35	11.16	7.24
Natural grassland (%)	19.64	32.65	28.58	36.91
Managed grassland (%)	0.00	0.92	0.00	3.42
Coironal (%)	12.04	17.11	9.49	11.51
Wetland (%)	5.15	1.97	3.30	1.42
Xerophytic scrub (%)	31.24	17.42	29.24	15.96
Mata Negra (%)	0.60	16.94	8.10	15.86
Mesophytic scrub (%)	4.15	2.54	4.22	4.30
Woods (%)	4.51	1.42	3.27	2.15

Values are expressed as percentages, except topographic and physiognomic variables.

TPNP: Torres del Paine National Park.

**Figure 4:**
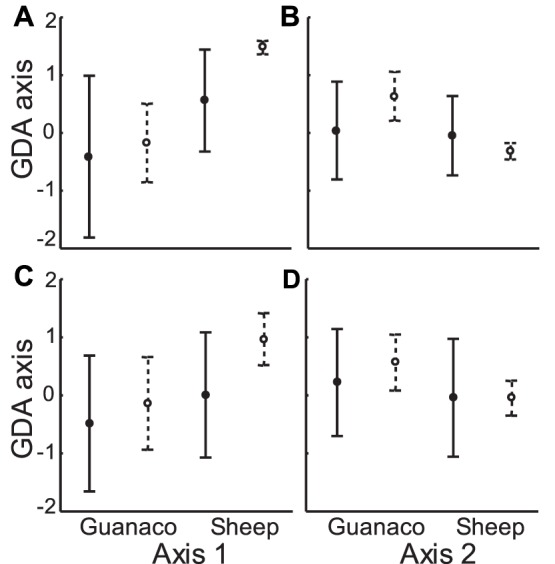
Habitats used by guanaco and sheep in winter. Means and standard deviations of canonical scores for preferred and marginal areas of guanaco and sheep in the first two axes defined by GDA in non-protected areas in winter 2009 (A, B) and winter 2010 (C, D). Filled circle and solid line represent marginal areas. Open circles and dotted line represent preferred areas.

Assessing possible overlap in winter between preferred and marginal areas, MANOVA test identified significant differences between preferred areas of guanaco and sheep during both years and on both GDA axes (F_2009_ = 13.51; d.f: 2,253; P<0.001; and F_2010_ = 5.79; d.f: 2,334; P = 0.003) (Fig. 4), while for marginal areas significant differences were found on axis 1, during both years (Fig. 4). Besides, guanaco marginal areas and sheep preferred areas showed significant differences during both winters, though only for axis 1 (Unequal-N HSD test, p<0.001).

### Summer

During summer, habitats selected by guanaco and sheep, as well as those described by the controls inside TPNP and non-protected area, were discriminated by the set of discriminant axes (canonical r = 0.520; p<0.001). Of the 6 significant axes, only the first two were chosen owing to their high share (69%) of discriminant power (Table 2). Topographic variables (slope -flat-, position –valley bottom- and roughness –low-) and three coverage types (bare soil, xerophytic scrub cover and mata negra) were the most important variables to discriminate among groups in the first axis (Table 3). In the second, the variables with the greatest weight were wetlands, woods and mata negra, and mean vegetation height (Table 3). As in winter, the first axis segregated the sites selected by guanacos and the habitat availability inside the TPNP from those selected by sheep and from the availability in the non-protected area (F_MANOVA_ = 94.56; p<0.001). The second axis discriminated between habitat selection of both species and environmental availability (F_MANOVA_ = 25.07; p<0.001).

**Figure 5:**
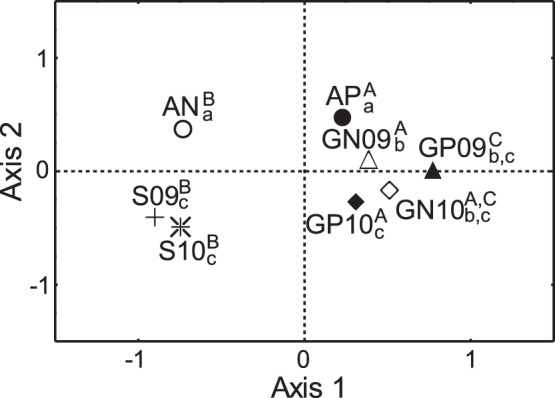
Results of the General Discriminant Analysis (GDA) in summer. Centroids of the observation groups are shown in the space defined by the first two discriminant axes of GDA. AP: Habitat availability in Torres del Paine National Park (TPNP); AN: Habitat availability in non-protected area; GP09: guanaco in TPNP in 2009; GP10: guanaco in TPNP in 2010; GN09: guanaco in non-protected area in 2009; GN10: guanaco in non-protected area in 2010; S09: sheep in non-protected area in 2009; S10: sheep in non-protected area in 2010. Different letters indicate significant differences between groups in first (superscript) and second axis (subscript) according to Unequal-N HSD *post-hoc* test (P<0.05).

Summer habitat availability (in terms of control sampling points in each zone; Table 4) was different between TPNP and non-protected areas (differences attributed to axis 1; Fig. 5). The sites used by sheep only showed significant differences with habitat availability in non-protected areas on axis 2, both in 2009 and 2010 (Fig. 5). In addition, they segregated of the sites selected by guanaco (inside TPNP and in non-protected area) on axis 1 in both years (Fig. 5). Sites used by guanaco in non-protected area did not differ between years, and inside the TPNP inter-annual differences were found only on the first axis (Fig. 5). Finally, sites selected by guanacos were significantly different from habitat availability in both areas and years (except in the case of guanacos in the TPNP in 2010, which showed significant differences with habitat availability only on axis 2; Fig. 5).

**Figure 6:**
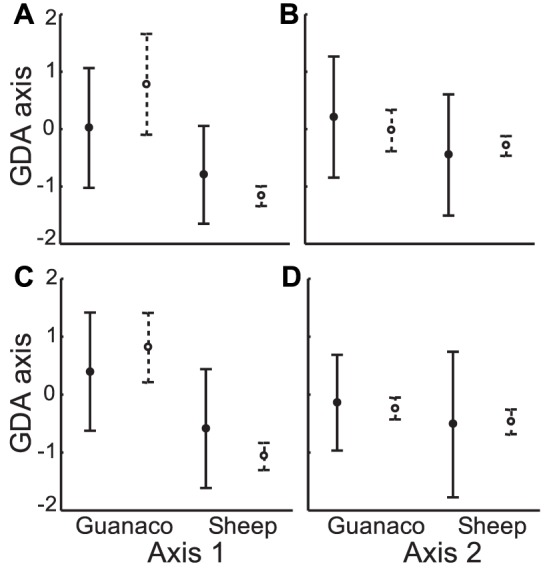
Habitats used by guanaco and sheep in summer. Means and standard deviations of canonical scores for preferred and marginal areas of guanaco and sheep in the first two axes defined by GDA in non-protected areas in summer 2009 (A, B) and summer 2010 (C, D). Filled circle and solid line represent marginal areas. Open circles and dotted line represent preferred areas.

Assessing possible overlap in summer between species in preferred and marginal areas, the emerging pattern displayed significant differences between guanaco and sheep in their preferred areas in both years restricted to the first axis (F_2009_ = 18.17; d.f: 2,387; P<0.001; and F_2010_ = 11.42; d.f: 2,452; P<0.001; Fig. 6). In the case of marginal areas, species differed in the two axes in 2009 and on the first axis in 2010 (Fig. 6).

## Discussion

The results show that habitat selection by guanaco in summer and in winter differs from sheep selection at a fine scale, in spite of their spatial aggregation at a landscape scale during three of the four seasons. In addition, this study allows a comparison of guanaco habitat selection in presence and absence of livestock, thanks to the presence of a control situation (inside TPNP) where the domestic species is absent. Thus, we found that guanaco did not modify its habitat selection in the presence of sheep, suggesting that the potential for competition between these species is low.

The spatial aggregation at landscape scale showed by guanaco and sheep, at least for three of the four seasons analysed, suggests the possibility for competition between both species at that scale. However, differential habitat use at a fine scale and selectivity in comparison with availability by the two species points to a low intensity of interaction between them in the present situation. Sheep displayed generalist behaviour, selecting in accordance with the available habitat, and, on a scale of tens of meters, habitats used by sheep differed from those of guanaco. On the contrary, habitats selected by guanacos differed from availability and they were similar both inside and outside the TPNP. These facts suggest a competition-free habitat selection by the wild herbivore.

The observed differences in habitat selection of co-occurring species have been attributed to different mechanisms: competitive displacement, plasticity or differential selection, among others [10], [37]. However, the absence of control situations in most studies makes it impossible to determine whether the apparent displacement of wild species towards suboptimal areas actually responds to some of them or to factors related to human activity difficult to quantify [38], [39]. It has been reported that large herbivores modify their behaviour, diet and/or habitat selection in the presence of livestock, trying to avoid it [14], [15], [16]. In this sense, a clear advantage of the present study is that it was possible to compare the guanaco habitat selection in both scenarios (with and without sheep) at one time and place. Guanaco showed a pattern of habitat selection consistent between years and seasons. According to previous studies, it selected open areas with abundant bare soil and small-size vegetation where a trade-off is reached between food availability and good visibility to reduce predation risk [19], [24], [40], [41]. This selection pattern was similar in TPNP and in non-protected area, indicating that guanaco did not modify its habitat selection despite the presence of livestock [39], [42], [43]. Nevertheless, the use of hillsides and sloppy terrain, rather than the flattest areas, could reflect some undetected factor that, at least in the non-protected area, may be associated with poaching or other human activity (see discussion below).

The low level of overlap between species in habitat selection at a fine scale shown here may be due to the fact that habitat diversity in the area allows them to make differential exploitation of available resources [37], [39]. Segregation at this scale was maintained throughout the year, even in winter, when the reduced availability of trophic resources could lead to a higher overlap in habitat use [39]. Segregation was found between guanaco and sheep both in preferred and marginal areas, as well as between guanaco marginal and sheep preferred areas. Hypothetically, this segregation pattern could be attributed to a high plasticity in resource use by guanaco, which could have shifted its realized niche towards habitats unused by sheep. However, the similarity between guanaco selection both inside and outside the protected area supports that guanaco did not modify its habitat selection due to livestock.

On the other hand, it does not appear that, in this case, the lack of interaction is due to low densities of the species, as suggested by Acebes et al [43]. In fact, the density of guanacos in the non-protected area (10.4 animals km^−2^; Iranzo et al, unpublished data) falls within the range described in other areas of Patagonia where these two species coexist [14], [44], [45], and the density of sheep is close to the sustainable stocking density in the area (Soto (SAG) pers. com.). This fact supports the idea that conditions are adequate in our study area to detect potential for competition between species, should this occur. Moreover, Patagonia has suffered an important desertification process as a consequence of the excessive density of sheep since the end of the 19^th^ century [22], [45], [46], [47]. A side effect of this has been a considerable loss of stocking capacity in the system, affecting the livestock production system as well as wildlife. In this context, studies such as this one, conducted in natural conditions with relatively high herbivore densities, are of particular interest in learning about the dynamics of ecosystems [43], [48].

This study, therefore, provides a new perspective: differences in habitat use at a fine scale by sympatric guanaco and sheep in Patagonia reflect a differential selection process. These results, based on habitat use, complement studies on diet that showed a high degree of coincidence between both species [23], [25], [26], and indicate that they may share trophic but not spatial resources at least under relatively high habitat heterogeneity. Nevertheless, it should be noted that habitat selection may be somewhat affected by pressure exerted by shepherds on the native species [38], [48]. Such harassment takes the form of poaching or persecution, which could lead guanacos to avoid some habitats and move to sub-optimal areas where human pressure is lower [14], [38], [43], [48]. Malo et al. [29] showed that the flight distance of guanacos in little-frequented areas was much greater that of those in more frequented areas, which points to a certain harassment effect from farmers and some degree of habituation to TPNP visitors by guanacos.

Finally, it must be taken into account that studies in natural conditions, such as this one, are subject to certain limitations that must be assumed as unavoidable since it is the only feasible way to study the interaction between large vertebrates, and the effects of livestock farming on wildlife [8], [14], [17]. These limitations are reflected, in this case, in certain differences in the proportions of available habitats inside the TPNP and the non-protected area, although this is not considered to have relevant impacts on the results obtained given consistency in guanaco habitat selection between zones and throughout the seasons and the vast area covered by the study.

In conclusion, the results show that there is low overlap between habitat used by guanaco and sheep at a fine scale, suggesting that, at present, there is low potential for competition between them. However, under different conditions, such as in sites with low habitat heterogeneity or with higher livestock pressure, as happened a few decades ago, the potential for competition may increase up to the triggering of demographic consequences on the species. Therefore, it would be interesting to further study the dynamics of the system in the face of potential future changes.

## Acknowledgments

We would like to thank CONAF (Corporación Nacional Forestal) and SAG (Servicio Agrícola y Ganadero, Ministerio de Agricultura, Chile) for their permission and support to conduct this study. Specially thanks to N. Soto and A. Kroeger for their collaboration and M.A. Vukasovic and N. Fuentes for their great help in the fieldwork. We are also grateful to the staff at the Torres del Paine National Park, ranchers from Torres del Paine municipality (XII Region, Chile), everyone else who helped us in fieldwork and the two anonymous reviewers who provided useful comments for the manuscript.
